# Exome Sequencing: Current and Future Perspectives

**DOI:** 10.1534/g3.115.018564

**Published:** 2015-07-02

**Authors:** Amanda Warr, Christelle Robert, David Hume, Alan Archibald, Nader Deeb, Mick Watson

**Affiliations:** * The Roslin Institute and Royal (Dick) School of Veterinary Studies, University of Edinburgh, Easter Bush, Edinburgh UK; † Genus plc., Hendersonville, Tennessee 37075

The completion of a reference genome sequence for humans took more than 200 scientists more than a decade in a project that cost almost $3 billion to complete (International Human Genome Sequencing Consortium 2004). During the past decade the price of genome sequencing has plummeted, much of the work has become automated, and methods have improved. These advances mean complete genomes can be sequenced quickly and affordably. Human genome sequencing is a special case and enjoys much lower cost-per-gigabase than other species because of Illumina’s HiSeq X platform (Watson 2014). Sequencing can allow for the identification of genetic variants that affect heritable phenotypes, including important disease-causing mutations and natural variation that can be exploited to improve crops and livestock. Despite the significant improvement in sequencing technology, sequencing whole genomes to a depth sufficient to find variants that affect phenotypic expression is expensive compared with targeted sequencing. This review will focus on exome sequencing, a method that targets only a subset of the genome, often the protein coding portion, significantly reducing the sequencing space and subsequently the cost. Details of the method, available platforms, uses in humans and other species, and its benefits over whole-genome sequencing (WGS) will be discussed.

## What Is Exome Sequencing?

The exome has been defined traditionally as the sequence encompassing all exons of protein coding genes in the genome and covers between 1 and 2% of the genome, depending on species. It also may be extended to target functional nonprotein coding elements (*e.g.*, microRNA, long intergenic noncoding RNA, etc.) as well as specific candidate loci. There are two main categories of exome capture technology: solution-based and array-based.

In solution-based, whole-exome sequencing (WES), DNA samples are fragmented and biotinylated oligonucleotide probes (baits) are used to selectively hybridize to target regions in the genome. Magnetic streptavidin beads are used to bind to the biotinylated probes, the nontargeted portion of the genome is washed away, and the polymerase chain reaction (PCR) is used to amplify the sample, enriching the sample for DNA from the target region. The sample is then sequenced before proceeding to bioinformatic analysis. Array-based methods are similar except that the probes are bound to a high-density microarray. The array-based method was the first to be used in exome capture (Albert *et al.* 2007), but it has largely been supplanted by solution-based methods, which require less input DNA and are consequently potentially more efficient; however, studies by Asan *et al.* (2011) and Bodi *et al.* (2013) found that NimbleGen’s Sequence Capture Array performed better than the solution-based alternatives in low GC content regions; had high sensitivity and read mapping rates; and single-nucleotide polymorphism (SNP) detection from these reads was more specific to the target region. This suggests that a niche may remain for the older technology. Array-based capture has been used successfully and accurately to identify rare and common variants and identify candidate genes for monogenic diseases in small cohorts (Ng *et al.* 2009); however, the array-based methods are less scalable owing to the limitation of the number of probes that can be accommodated on the array and additional equipment and time required to process the microarrays.

## Exome Capture Platforms

There are several differences between the available platforms, which are constantly being updated and improved. The major providers of exome capture platforms are NimbleGen, Agilent, and Illumina, and each have different designs and strengths (summarized in Table 1). The discussion of the characteristics of each platform will focus on the solution-based human kits; the performance of kits for other species has not been subjected to the same level of comparison.

**Table 1:** Summary of the differences between the solution-based, exome-sequencing platforms

	NimbleGen’s SeqCap EZ Exome Library	Agilent’s Sure Select Human All Exon Kit	Illumina’s TruSeq Exome Enrichment Kit	Illumina’s Nextera Rapid Capture Exome Kit
Probe size, bp*^a^*	55−105	114−126	95	95
Probe type	DNA	RNA	DNA	DNA
Coverage strategy	High-density, overlapping probes	Adjacent probes	Gaps between probes	Gaps between probes
Fragmentation method	Ultrasonication	Ultrasonication	Ultrasonication	Transposomes
Target region size (human), Mb*^b^*	64	50	62	62
Reads remaining after filtering*^c^*	66%	71.7%	54.8%	40.1%
Major strengths	(i) High sensitivity and specificity	(i) Better coverage of indels	(i) Good coverage of UTRs and miRNAs	(i) Good coverage of UTRs and miRNAs
	(ii) Most uniform coverage in difficult regions	(ii) High alignment rate		
		(iii) Fewer duplicate reads than other platforms		
Major weaknesses	(i) More duplicate reads than Agilent	(i) Fewer high-quality reads than NimbleGen	(i) High off-target enrichment	(i) High off-target enrichment
	(ii) Lower alignment rate than Agilent			(ii) Coverage bias for high GC content areas reducing uniformity
Nonhuman supported	Yes	Yes	No	No

UTR, untranslated region; miRNA, microRNA.

a As described in Clark *et al.* (2011), NimbleGen SeqCap EZ v2.0, Agilent SureSelect Human All Exon 50Mb, Illumina TruSeq Exome Enrichment.

b For NimbleGen SeqCap EZ v3.0, Agilent SureSelect V5, and Illumina TruSeq and Illumina Nextera original versions.

c Filtering for duplicates, multiple mappers, improper pairs, and off-target reads, data from Chilamakuri *et al.* (2014) .

NimbleGen’s SeqCap EZ Exome Library has the greatest bait density of any of the platforms and uses short (55−105 bp), overlapping baits to cover the target region (Clark *et al.* 2011). This approach has been found to be an efficient method for enrichment with the least amount of sequencing needed to cover the target region and sensitively detect variants (Clark *et al.* 2011) and also has a high level of specificity, showing fewer off-target reads than other platforms (Clark *et al.* 2011; Sulonen *et al.* 2011). Importantly, this bait design has been found to show greater genotype sensitivity and more uniformity of coverage in difficult to sequence regions, such as areas of high GC content, than the other platforms (Asan *et al.* 2011; Sulonen *et al.* 2011; Bodi *et al.* 2013).

Agilent’s SureSelect Human All Exon Kit is the only platform to use RNA probes, with the other platforms opting for DNA probes. The baits used are longer than those used in NimbleGen’s platform (114−126 bp) and the corresponding target sequences are adjacent to one another rather than overlapping (Clark *et al.* 2011). This design has been found to be good at identifying insertions and deletions (indels), because longer baits can tolerate larger mismatches (Clark *et al.* 2011; Bodi *et al.* 2013; Chilamakuri *et al.* 2014); it has been suggested that this also may reduce reference allele bias at heterozygous sites compared with other bait designs; however, in practice the allele bias has been similar to other platforms (Asan *et al.* 2011; Hedges *et al.* 2011). The platform has been found to produce fewer duplicate reads than NimbleGen but also fewer high-quality reads (Sulonen *et al.* 2011). Bodi *et al.* (2013) found that Agilent had a greater alignment rate and fewer PCR duplicates than NimbleGen but also had less uniform coverage.

Illumina’s TruSeq Exome Enrichment Kit uses 95-bp probes that leave small gaps in the target region, with paired end reads extending outside the bait sequence during sequencing to fill the gap. This design has been found to have a high percentage of off-target enrichment (Clark *et al.* 2011), which reduces its target efficiency compared with the other platforms. This kit detects more single-nucleotide variants in the untranslated regions (UTRs) than the other platforms (Clark *et al.* 2011), although comparisons of performance with NimbleGen and Agilent’s “+UTR” kits have yet to be performed. Following filtering for duplicates, multiple mappers, improper pairs, and off-target reads, Chilamakuri *et al.* (2014) found that this platform retained fewer reads (54.8%) than NimbleGen (66%) or Agilent (71.7%). At high read counts (>50M), this platform outperformed Agilent’s SureSelect in downstream identification of indels (Clark *et al.* 2011).

Illumina’s Nextera Rapid Capture Exome and Expanded Exome kits are similar to the TruSeq kit in their probe design. They differ from the other kits in that they use transposomes to fragment the genomic DNA, whereas the other platforms use ultrasonication. These kits have not been extensively compared with the other platforms, with only Chilamakuri *et al.* (2014) having included Nextera in a comparison study. At the time of the study there was only one Nextera kit, which was the Expanded Exome version: the kit with the larger target region of the two. The Expanded Exome kit shares a target region with the TruSeq kit, which includes UTRs and miRNAs. Chilamakuri *et al.* (2014) found that the Nextera kit had increased coverage of high GC content areas as the result of altered bias in the transposome technology used during fragmentation, decreasing its overall uniformity; however, recent changes to the protocol in the new versions may have improved this. They also found that out of all platforms tested, the Nextera platform retained the fewest reads after filtering for duplicates, multiple mappers, improper pairs, and off-target reads, at 40.1%.

Chilamakuri *et al.* (2014) compared the target regions of the human kits from three providers (NimbleGen SeqCap EZ v3.0 – 64.1Mb; Agilent SureSelect V4 – 51.1Mb; and Illumina TruSeq and Illumina Nextera original version and protocol – 62.08 Mb). Despite the fact that all of the platforms are targeting the human exome, there is surprisingly little overlap between the three designs, with just 26.2 Mb covered by all three target regions. NimbleGen’s and Agilent’s coverage are more similar to one another than either are to Illumina’s; this is likely attributable to the large amount of UTRs covered in Illumina’s target region. Illumina has 22.5Mb of targets unique to their platforms, and 21.8Mb of these are UTRs. NimbleGen and Agilent have 16.1 Mb and 7 Mb of unique targets, respectively. It would be interesting to see how NimbleGen and Agilent’s “+UTR” kits compare with Illumina in single-nucleotide variant calling, particularly in UTRs.

Currently, in addition to human kits, NimbleGen offers capture kits for maize, barley, wheat, soy, mouse, and pig exomes, and Agilent offers capture kits for mouse, cattle, and zebrafish exomes. Both providers also offer the opportunity to design custom kits for other species. The kits for nonhuman species use similar bait designs and protocols to the providers’ human kits. Both manufacturers offer a flexible design process that allows for modifications to improve coverage for specific regions and purposes.

## Uses in Humans

The first successful use of WES to diagnose and inform subsequent treatment in a human patient was in the identification of the causal variant of a rare form of inflammatory bowel disease in an infant (Worthey *et al.* 2011). In this case, conventional diagnostics had failed to find an explanation for the patient’s severe symptoms, and doctors needed to understand the underlying cause of the symptoms before they could decide how to treat the child. A multidisciplinary team combined clinical phenotyping, exome sequencing, bioinformatics, and functional studies, eventually finding the causative mutation that influenced the future treatment of the child. The analysis of the exome data was hampered by the relative lack of software designed for this purpose at the time, and researchers had to manually inspect more than 2000 variants. Through filtering and manual inspection, the candidate pool was reduced to 70 genes exhibiting hemi- or homozygous variants. Of these, only eight variants were novel and predicted to be damaging to protein function. Analysis of evolutionary conservation identified two variants that were located in highly conserved sequences and one of these had a high null genotype frequency. This left a single hemizygous, nonsynonymous variant in the *X-linked inhibitor of apoptosis (XIAP)* gene. The child was diagnosed with X-linked lymphoproliferative disease 2, exhibiting a novel inflammatory bowel disease−like manifestation caused by loss of tolerance to commensal organisms in the digestive system. This diagnosis allowed for effective treatment through allogeneic hematopoietic progenitor cell transplant. The unusual manifestation of the condition meant that the patient was unlikely to have been diagnosed without the exome sequence data.

After the success of this initial diagnosis, exome sequencing has been used extensively to diagnose novel diseases and find novel causative mutations for known disease phenotypes. Exome sequencing is useful in human medicine for diagnosis of particularly difficult-to-diagnose patients, diagnosis of young patients who may not yet exhibit a full spectrum of symptoms (Iglesias *et al.* 2014), prenatal diagnosis (Iglesias *et al.* 2014; Xu *et al.* 2014), and early diagnosis of debilitating disease (Bras and Singleton 2011; Sassi *et al.* 2014). In addition to reaching a diagnosis, finding the causative mutation can allow for alteration of treatment, prevention of further invasive testing, accurate prognoses, and confirmed diagnoses, which are essential for eligibility for benefits and access to clinical trials (Grossmann *et al.* 2011; Rabbani *et al.* 2012; Taneri *et al.* 2012; Iglesias *et al.* 2014) and in the future may allow for more targeted treatment.

Exome sequencing in human medicine benefits from the availability of large databases of known SNPs, known pathogenic variants, and control genomes; during analysis variants found in these databases can generally be excluded when one is looking for novel variants, significantly reducing the variant pool. The Exome Aggregation Consortium (ExAC) has created a user-friendly database containing the exome sequences of more than 60,000 unrelated individuals, which is freely available (Exome Aggregation Consortium (ExAC) 2014). The exomes have been analyzed using a uniform bioinformatic pipeline and are from individuals with adult-onset diseases. This provides researchers with a large set of reference exomes that should be free from homozygous variants that cause childhood-onset Mendelian diseases. The database provides a wealth of information such as depth of coverage, genotype quality, allele frequency, and variant consequences. The filtering capabilities and large number of exomes will simplify the process of prioritizing variants when using exome sequencing as a diagnostic tool, particularly in children.

Where many unrelated, affected individuals are available, an “overlap” strategy can be used to simplify analysis and search for common variants likely to affect gene function (Johansson *et al.* 2012). If there is a known inheritance pattern this can be used to search for specific genotype zygosity. Sequencing of multiple affected related individuals also can increase the power of the analysis (Johnson *et al.* 2010; Shi *et al.* 2011). In rare diseases, case−parent trios can be used to exclude nonpathogenic variants found in the parents (Smith *et al.* 2014). However, single-patient sequencing may be sufficient to identify the causative mutation (Wang *et al.* 2011; Worthey *et al.* 2011; Xu *et al.* 2014). Analysis examining how conserved an amino acid sequence is through evolution and between genes in a family can help to increase the confidence of a mutation having a deleterious effect (Wang *et al.* 2011; Johansson *et al.* 2012; Sassi *et al.* 2014; Smith *et al.* 2014; Xu *et al.* 2014).

Examples of diseases for which exome sequencing has been used to detect a causative variant include Leber congenital amaurosis (Wang *et al.* 2011), Alzheimer disease (Sassi *et al.* 2014), maturity-onset diabetes of the young (Johansson *et al.* 2012), high myopia (Shi *et al.* 2011), autosomal recessive polycystic kidney disease (Xu *et al.* 2014), amyotrophic lateral sclerosis (Johnson *et al.* 2010), immunodeficiency leading to infection with human herpes virus 8 causing Kaposi Sarcoma (Byun *et al.* 2010), acromelic frontonasal dystois (Smith *et al.* 2014), and a number of cancer predisposition mutations (*e.g.*, Yan *et al.* 2011; Greif *et al.* 2012; Snape *et al.* 2012; Kiiski *et al.* 2014; Cai *et al.* 2015).

The Deciphering Developmental Disorders project aims to develop a scalable exome sequencing workflow to facilitate the translation of the method from the research environment to a clinical environment. So far more than 1000 children with undiagnosed developmental disorders and their parents have been sequenced with plans to increase this number to 12,000 patients. With diagnostic yields up to 31% in the children sequenced thus far, this project demonstrates the future potential of the method in a clinical setting (Gecz and Corbett 2015; Wright *et al.* 2015; The Deciphering Developmental Disorders 2015).

## Uses in Other Species

### Variants discovery for agricultural improvement

Not all protein-altering variants cause disease (Macarthur and Tyler-Smith 2010). To understand the potential of exome sequencing in areas other than disease variant discovery, which is the main focus in human research, we can look to studies using the technique to sequence agricultural species.

Plant genomes can be extremely complex, repetitive, and are often polyploid; as a result, some of the most economically important crops are not well suited for genome re-sequencing studied. For example, Bread Wheat (*Triticum aestivum*) has an allohexaploid (AABBDD) genome around 17Gb in size (Brenchley *et al.* 2012; The International Wheat Genome Sequencing Consortium 2014). This is likely too large for subsequent WGS studies at the current price of sequencing and data storage. Wheat is an extremely important crop for both human and livestock consumption and genetic improvement is slow; with the human population increasing and additional challenges from environmental changes, it is essential to gain a better understanding of the crop to improve it. An exome capture kit has been designed for wheat based on the accumulated transcriptome data (Winfield *et al.* 2012). The capture region for this kit is 56.5 Mb, which is around the lower estimated size of one diploid wheat exome and because of similarity between the three genomes may be sufficient to capture most of the exome data from the whole allohexaploid genome. This kit has been used to identify induced mutations in the genome to aid studies investigating gene function, a use that also was applied to the rice (*Oryza sativa*) exome in the same study (Henry *et al.* 2014) and the soybean (*Glycine max*) exome in a separate study (Bolon *et al.* 2011). WES in soybean has also been used to identify unwanted intracultivar genetic heterogeneity in the exome that may affect the plant’s phenotype (Haun *et al.* 2011).

The genome of barley (*Hordeum vulgare* L.) has not been fully sequenced. Barley’s genome is smaller than wheat’s at around 5 Gb, but is still larger than is practical for routine whole-genome sequence analysis and contains repetitive elements that complicate the genome’s assembly. A gene space assembly has been produced (The International Barley Genome Sequencing Consortium 2012) and a barley exome capture kit has been developed based on this assembly (Mascher *et al.* 2013). The kit has since been used to identify a mutation involved in early maturation, a trait relevant to production (Pankin *et al.* 2014).

Exome capture in barley has also been used to identify a gene causative of many-noded dwarfism using mapping-by-sequencing (Mascher *et al.* 2014). The many-noded dwarfism phenotype is a shorter plant with more, narrower leaves than the wild type. The mutant in this study was created using X-ray mutagenesis, a technique that often causes large deletions. An F_2_ population between mutant and wild-type phenotypes was created and 18 mutant individuals and 30 wild-type individuals were exome sequenced. From these sequences SNPs were identified and allele frequencies of these were used to identify an allele overrepresented in the mutant group. Researchers queried the sequencing reads for exome targets that were present in the wild type but not the mutant. This lead to the identification of a candidate gene (MLOC_64838.2, now HvMND), which has a homolog known to play a role in a similar phenotype in rice. Screening of other mutants showing this phenotype found a variety of null mutations in this gene. The family to which this gene belongs is known to have effects on important production traits and may include good selection targets to improve production.

### Identification of new genetic markers

Robert *et al.* (2014) have designed exome capture probes for the pig *Sus scrofa*, used these to sequence the exomes of 96 healthy pigs, and identified potentially deleterious variants. Bioinformatic analysis identified 236,608 high-confidence predicted variants and 28,115 predicted indels in the target region. This work revealed notable gaps in the current Ensembl *S.scrofa* genome annotation and identified a large number of potential protein truncating variants. As the pigs tested were healthy, it is possible that some of these protein truncating variants have phenotypic effects on traits other than those relating to the health of the pigs or to the production traits currently under selection. This work is an important step in identifying phenotype altering variants in the pig: a production animal and medical model.

The barley exome kit has been used to differentiate between markers of *H. vulgare* L. and *H. bulbosum* L.; *H. bulbosum* L. is a wild species that has superior pathogen resistance and tolerance compared to the domestic species and the two can be crossed to improve the domesticated crop, however negative linkage drag on production traits has hampered its use in elite barley lines. Using exome sequencing to identify specific markers can allow selective crossing to be used to incorporate the beneficial variants without incorporating linked variants that are detrimental to production (Wendler *et al.* 2014). Similarly, the wheat exome kit has allowed for discovery of previously unidentified markers in the genome which can be used in future genetic studies and marker assisted selection (Allen *et al.* 2013).

Black cottonwood (*Populus trichocarpa*) has had an exome capture kit designed (Zhou and Holliday 2012). *P. trichocarpa* is a model organism and was the first tree to have its whole genome sequenced (Tuskan *et al.* 2006). The tree is used in lumber production and in cosmetics. The tree has experienced a whole genome duplication fairly recently and the exome study found that this does not appear to have had an effect on SNP detection through exome sequencing (Zhou and Holliday 2012). There is potential to use the identified markers to improve production in this species.

### Health traits

As with exome sequencing in humans, exome sequencing has been used in other mammals to discover variants associated with health traits. WES has been used in conjunction with a genome wide association study to identify a frameshift mutation causing blindness in Phalène dogs (Ahonen *et al.* 2013). In cattle (*Bos taurus*), WES has been used successfully to identify strong candidate variants for haplotypes relating to reduced fertility rates in Holsteins which can be used to selectively breed against these detrimental haplotypes (McClure *et al.* 2014).

### Special considerations for nonhuman species

For many species, the reference genome is not completed to the same standard as the human genome; many species have only a draft genome or, as discussed for barley, no reference genome. This is an important consideration when using WES in nonhuman species. Poor annotation of genomes mean that in the design of capture probes, causative genes may be missed because they are not annotated whereas with whole genome sequencing, the data will be there whether the gene is annotated or not. Where available, including predicted genes identified from RNA-sequencing data may be beneficial to maximize coverage of functional elements in poorly annotated genomes. For example, Robert *et al.* (2014) added an additional 14Mb of data to the capture region in pigs using EST evidence. Additionally, errors in the reference genome will greatly increase the number of false-positive variant calls in these species. This increases computational burden and forces more stringent filtering which may inadvertently discard causative variants.

WES may be especially useful for model organisms, particularly where the sequences of large numbers of individuals are needed. The expense of WGS on large cohorts makes it less feasible in animal studies than human studies, particularly in species with fairly large genomes such as mice (∼3.5 Gb) and zeberafish (∼1.5 Gb), both common model organisms. Sequencing a smaller portion of the genome, particularly if candidate regions are known and can be targeted, would be more cost-effective, allow deeper coverage and potentially increase the number of individuals that can be used in these studies.

The genomes of animals have different levels of linkage disequilibrium (LD), which are often high in domestic species and model organisms with limited effective population sizes. The level of LD also may vary by breed, as demonstrated in the domestic dog (Stern *et al.* 2013). When using exome sequencing to identify causal variants, high LD may lead to the identification of a benign variant that is in LD with the causative variant. In this case, the causative variant may have been missed in variant calling or lie outside the sequence space; it is therefore important to consider the known function of the element the candidate variant is associated with and the predicted effect of the variant on function. Additionally, efforts to sample individuals that are as outbred as possible may help to reduce this problem. Another consideration with high LD is that it may be possible to use this information in imputation, as has been done with other technologies such as imputing genotypes from SNP arrays (Hickey *et al.* 2012), from WGS data (Deelen *et al.* 2014; Gudbjartsson *et al.* 2015) and from human exome data (Auer *et al.* 2012). The larger haplotypes associated with high LD increase the accuracy and reduce the computational burden associated with imputation. Imputation may be less accurate than sequencing; however it allows for a larger numbers of individuals to be used at lower coverage and may help to reduce problems associated with variable coverage in exome sequencing.

## Exome Capture Transferability Between Species

The Neanderthal exome was successfully sequenced by Burbano *et al.* (2010) using an exome capture kit designed for the human exome. The study compared the Neanderthal exome with human exomes and found 88 fixed substitutions in 83 genes in the human exomes; these substitutions did not appear to be a result of positive selection and may be a result of accelerated genetic drift from reduced effective population size in humans following historical bottlenecks or reduced purifying selection.

The kit designed for cattle also can be applied to other bovid species; Cosart *et al.* (2011) demonstrated that the kit could be successfully used to capture the exomes of, and identify SNPs in, zebu (*Bos indicus*) and American Bison (*Bison bison*). This transferability of exome capture kits, as also demonstrated in studies involving the sequencing of Neanderthals and nonhuman primates using human capture kits (Burbano *et al.* 2010; Vallender 2011), is possible because despite millions of years of divergence, functional elements tend to be highly conserved.

## Why Not Use Genome Sequencing?

With the price of sequencing decreasing as rapidly as it has during the past decade, questions have been raised concerning WES’s usefulness in the era of affordable WGS. WGS, however, is still more expensive than WES. The costs of WES consist of the cost of the capture plus the cost of sequencing, whereas WGS consists only of the sequencing costs. If we assume that the cost of capture remains fixed, then as the costs of sequencing decrease, the cost of WGS will approach the cost of WES. However, at present that is not the case, and it would be unwise to assume that the cost of sequence capture will not decrease.

Human genomes are a special case in that the HiSeq X platform offers a cost-per-Gb far less than other platforms yet is limited (contractually, rather than technically) to 30X WGS human genomes (Watson 2014). However, even given that advantage, the $1000 price tag for a 30X human genome is (in our estimate) two to three times the cost of a 40X human exome (depending on scale). It may be advantageous to sequence more samples using WES, and gain statistical power, than to sequence more of the genome. In other species, the price difference is even greater, for example, in pigs (a similar sized genome to human but currently without the benefit of access to the HiSeq X platform) we estimate the cost of WGS to be 9-10 times the cost of WES (Robert *et al.* 2014).

Although WGS does have benefits over WES, the cost of this technology is more than simply the price of sequencing. Although sequencing technology has been improving at a much faster rate than would be predicted by Moore’s law (a prediction of improvement in computing hardware, but often also applied to other technologies), the technology for storing and analyzing the data has not seen a matching acceleration in improvement (Mardis 2010; Sboner *et al.* 2011). WGS produces approximately one hundred times the data that WES does at the same coverage. The infrastructure needed to store, manage, and analyze data significantly increases the costs of WGS. WGS produces a much larger number of variants than WES does, not only because of the size of the sequencing space, but because regions outside the exome are less well conserved; although this number might include a variant of interest, the larger data set significantly increases the computational burden for analysis. Additionally variation in noncoding regions is less well understood than variation in the coding region (Mu *et al.* 2011; Maurano *et al.* 2012; Ward and Kellis 2012), making it more difficult to predict which variants might be relevant to a trait of interest in WGS datasets. The majority of causative variants identified so far in Mendelian disease have been found in coding regions (Botstein and Risch 2003), although ascertainment bias is likely to play a role in this conclusion. A study investigating functional noncoding variants based on WGS data from 1092 human genomes showed that functionally deleterious non-coding mutations were under strong negative selection, in a similar way to that of loss-of-function variants in protein-coding regions (Khurana *et al.* 2013). The authors developed a tool to prioritize non-coding variants in disease studies, which was used to identify noncoding candidate drivers in tumor genomes.

With the continuous decrease in sequencing cost, new studies making use of WGS to investigate causative variants will lead to the discovery of additional mutations in regulatory elements that contribute to the pool of disease-associated variants. In that context, the sampling bias currently observed toward coding variants is likely to be reduced by WGS investigations of non-coding genomic regions.

However, cost is not the only consideration. WGS covers the whole genome at more consistent coverage than WES, can provide more accurate detection of structural variants, and does not have reference sequence bias caused by probe sequences in WES (Majewski *et al.* 2011; Meynert *et al.* 2014; Belkadi *et al.* 2015). Recent studies have highlighted and suggested roles for promoters (The Fantom Consortium and the RIKEN PMI and CLST 2014) and enhancers (Andersson *et al.* 2014) in a range of different cell types, and these are not traditionally captured by exome sequencing. Importantly, WES requires previous knowledge of the location and sequence of features to target them, whereas WGS covers the entire genome. This means that exome sequencing relies on the accuracy of the genome annotation, so phenotype altering variants may be missed in poorly or incompletely annotated genomes. It is therefore important to take care when designing the capture region or purchasing a commercial kit to ensure any specific regions of interest are included. Exome sequencing also invariably fails to successfully capture the entire target region (Asan *et al.* 2011; Bodi *et al.* 2013; Chilamakuri *et al.* 2014; Robert *et al.* 2014), causing even properly annotated regions to be missed. Another consideration is that exome sequencing involves a PCR stage that is known to reduce coverage of GC-rich regions (Kozarewa *et al.* 2009; Veal *et al.* 2012). New sequencing technology has allowed for sequencing of DNA without the need for DNA amplification, generating sequencing from single molecules. This technology can produce accurate, longer reads without the artifacts and biases associated with the amplification process in other sequencing methods and the exome capture stage of WES (Shin *et al.* 2013). The longer read length in these third-generation sequencers is particularly beneficial for detecting structural variants and for resolving repetition in assemblies and copy number variable regions (Roberts *et al.* 2013). However, for now the price of this long-read sequencing is still prohibitively expensive and is not commonly in use for analysis of genetic variation. In the future as these sequencers improve and prices come down they may make WGS more attractive to researchers even if their analysis focuses solely on the protein-coding region and known functional elements.

The power of WGS on a large cohort for variant discovery already has been demonstrated by Gudbjartsson *et al.* (2015), who sequenced the whole genomes of 2636 Icelanders to 20X and imputed the sequence variants into 101,584 further chip-genotyped and phased individuals from the same population. This allowed for discovery of 6795 null mutations in 4924 genes that may play a role in disease. They also found evidence of lethal mutations that are not found in the homozygous state, which likely impact on fertility in heterozygous couples. Imputation reduces the amount of sequencing and data storage required, though the study found that fewer null mutations were detected in the imputed data set suggesting that some mutations were missed in these individuals.

WGS will eventually take a leading role in genome interrogation; however, it will likely have to wait for data storage and analysis to improve before its full potential can be realized. In the meantime, WES provides many of the benefits of WGS with lower storage requirements and computational burden, at an affordable price. This is particularly useful in large scale studies, for example, a recent study sequenced the exomes of more than 9000 people (Schick *et al.* 2015). It is also useful for the sharing of information such as in the ExAC database where there are more than 60,000 exomes stored. WES will likely also remain the method of choice in species with exceptionally large genomes, for example in some polyploid plant species.

Exome sequencing is a technology that allows interrogation of the most well-understood portion of the genome: the protein-coding sequence and functional elements. Variants of interest don’t necessarily fall within the exome, but so far most of the known variants responsible for Mendelian disease have been found in the coding region and the target region can be extended to include other regions of interest. Although the decreasing price of sequencing may soon make genome sequencing more attractive, the additional costs of data handling and downstream analysis cannot be ignored. The applications of variant discovery, particularly in disease gene identification, cannot afford to wait for data storage and processing technology to catch up to sequencing improvements. The amount of data produced by WES is far more manageable than WGS, particularly for small research groups and groups studying organisms with large genomes. WES has established itself as an important method in disease gene identification in humans and increasingly in domestic species. The applications of WES in crop research are allowing genomic techniques to be used in species with complex genomes, potentially identifying variants important for production that can be incorporated into marker-assisted selection. At present, WES is a useful and powerful method for variant discovery within coding regions offering most of the benefits of WGS while allowing for easier analysis and storage of the data produced.

However, WGS will eventually be the NGS technology of choice with regard to the investigation of genomic variations due to the more uniform coverage achieved with WGS *vs*. WES, including coverage within the exome (Belkadi *et al.* 2015) and the more uniform distribution of sequencing quality parameters (*e.g.*, coverage depth, genotype quality) observed with WGS *vs.* WES (Meynert *et al.* 2014; Belkadi *et al.* 2015). In addition, WGS extends the variation search space to the whole genome, allowing for the additional detection of noncoding variants (The 1000 Genomes Project Consortium 2012), the functional impact of which is becoming easier to interpret with the systematic annotation of functional noncoding elements (The Encode Project Consortium 2012; Ward and Kellis 2012; Andersson *et al.* 2014; The Fantom Consortium and the RIKEN PMI and CLST 2014).

Ideally, regardless of sequencing costs and storage limitations, integrated approaches combining the advantages of both WES and WGS would be beneficial for variant discovery studies with the addition of WES-/WGS-exclusive variants with WES providing additional variants missed in low-coverage dataset (The 1000 Genomes Project Consortium 2012). In addition, both technologies’ usefulness depend on the quality of the reference assembly that the sequenced reads are mapped to; poor quality references increase the number of false-positive variants identified in the analysis, which inevitably leads to more stringent filtering, increasing the potential for discarding a variant of interest. In the future, the improvement of reference assemblies, bioinformatic tools and sequencing technology will be necessary to improve the power of variant discovery techniques.

The term “exome” may no longer be appropriate for a technique that is simply a subset of the more generic technique of sequence capture. It is already possible to extend the exome capture region beyond protein coding genes to capture noncoding genes, and regulatory elements such as promoters and enhancers. In this way, we may sequence all of the functional areas of a genome without the cost of sequencing everything. Indeed, as clinically important variants are discovered, the paradigm may change to sequencing small panels of genes that are known to be relevant to disease, as is common in cancer genomics.
